# Impaired Nicotinamide Adenine Dinucleotide Biosynthesis in the Kidney of Chronic Kidney Disease

**DOI:** 10.3389/fphys.2021.723690

**Published:** 2021-09-17

**Authors:** Xinhui Liu, Denggui Luo, Shiying Huang, Siqi Liu, Bing Zhang, Fochang Wang, Jiandong Lu, Jianping Chen, Shunmin Li

**Affiliations:** ^1^Department of Nephrology, Shenzhen Traditional Chinese Medicine Hospital, Guangzhou University of Chinese Medicine, Shenzhen, China; ^2^Shenzhen Key Laboratory of Hospital Chinese Medicine Preparation, Shenzhen Traditional Chinese Medicine Hospital, Guangzhou University of Chinese Medicine, Shenzhen, China

**Keywords:** chronic kidney disease, nicotinamide adenine dinucleotide, the *de novo* pathway, the salvage pathway, the Preiss-Handler pathway

## Abstract

Chronic kidney disease (CKD) is a global public health problem with high morbidity and mortality. Decreased nicotinamide adenine dinucleotide (NAD^+^) levels were found to be associated with aging, cancer, and neurodegenerative and metabolic disorders. However, the alteration of renal NAD^+^ levels and biosynthesis pathways in CKD is less known. In the present study, we aimed to evaluate renal NAD^+^ levels and tested the expression of key enzymes in three NAD^+^ biosynthesis pathways in two different types of CKD rat model. CKD rat models were established by 5/6 nephrectomy (5/6 Nx) and feeding with adenine-containing feed, respectively. Renal function was assessed by serum creatinine (Scr) and blood urea nitrogen (BUN). Renal pathology was evaluated by periodic acid-Schiff (PAS) and Masson’s trichrome staining. The expression of key enzymes in three NAD^+^ biosynthesis pathways was determined and quantified by Western blot analysis. The results showed CKD rat models were successfully established as evidenced by increased Scr and BUN levels, upregulation of neutrophil gelatinase-associated lipocalin (NGAL), glomerular hypertrophy, and renal fibrosis. Renal NAD^+^ and NADH content were both declined in two CKD rat models, and NAD^+^ levels were negatively correlated with Scr and BUN levels in CKD rats. Three key enzymes involved in NAD^+^ biosynthesis were significantly downregulated in the kidney of both of the two CKD models. They were quinolinate phosphoribosyltransferase (QPRT) in the *de novo* pathway, nicotinamide mononucleotide adenylyltransferase 1 (NMNAT1), and NMNAT3 in the salvage pathway. Moreover, the expression of NAD^+^-consuming enzymes sirtuin 3 (SIRT3) and CD38 decreased significantly in CKD rats. In conclusion, NAD^+^ biosynthesis was significantly impaired in CKD, which may attribute to downregulation of QPRT and NMNAT 1/3.

## Introduction

Chronic kidney disease (CKD) is becoming a public health problem worldwide. The global estimated prevalence of CKD is 13.4% (11.7–15.1%; Lv and Zhang, 2019). Regardless of the primary disease, CKD is slowly progressive and leads to end-stage kidney disease (ESKD) and premature death (Ruiz-Ortega et al., 2020). CKD is the 16th leading cause of years of life lost worldwide (Chen et al., 2019). Therefore, it is urgent to study the pathophysiological mechanism of the development and progression of CKD.

Nicotinamide adenine dinucleotide (NAD) exists in two forms, including an oxidized (NAD^+^) and a reduced (NADH) form (Zapata-Perez et al., 2021). NAD was originally discovered by Harden and Young in 1906 as a coenzyme involved in yeast fermentation (Harden and Young, 1906). Since then, multiple roles of NAD^+^ in cell signaling and survival pathways have been discovered. Decreased NAD^+^ levels were found to be associated with aging, cancer, and neurodegenerative and metabolic disorders (Amjad et al., 2021). In mammals, NAD^+^ is synthesized through three pathways: (1) the *de novo* pathway from tryptophan (Trp), (2) the Preiss-Handler pathway from nicotinic acid (NA), and (3) the salvage pathway from nicotinamide (NAM), a by-product of NAD^+^-consuming enzymes such as sirtuins, poly (ADP-ribose) polymerase (PARP), and CD38, or nicotinamide riboside (NR; Yaku et al., 2018). The renal tubular epithelial cells are highly metabolically active and require a constant supply of ATP to provide the energy for reabsorption and regulation of diverse cellular processes. NAD^+^ has critical role in the generation of ATP and as a substrate for important enzymes that regulate cellular health and stress responses (Ralto et al., 2020). Therefore, NAD^+^ biosynthesis is essential for maintaining kidney function. Recently, Poyan Mehr et al. (2018) found that impaired *de novo* NAD^+^ biosynthesis caused by impaired quinolinate phosphoribosyltransferase (QPRT) exacerbated acute kidney injury (AKI) susceptibility. Katsyuba et al. (2018) showed that inhibition of α-amino-β-carboxymuconate-ε-semialdehyde decarboxylase (ACMSD), the enzyme that limits spontaneous cyclization of α-amino-β-carboxymuconate-ε-semialdehyde in the *de novo* NAD^+^ biosynthesis pathway, increased NAD^+^ levels in a tissue-specific manner and provided protection in models of AKI and non-alcoholic fatty liver disease. These two studies highlighted the importance of *de novo* NAD^+^ biosynthesis in AKI. However, the alteration of renal NAD^+^ levels and biosynthesis pathways in CKD is less known. In the present study, we established two different types of CKD rat model, measured renal NAD^+^ levels, analyzed the correlation between NAD^+^ levels and renal function indexes, and tested the expression of key enzymes in three NAD^+^ biosynthesis pathways.

## Materials and Methods

### Animals

All animal experiments were conducted with protocols approved by the Experimental Animal Ethics Committee of Guangzhou University of Chinese Medicine. Male Sprague Dawley (SD) rats weighted 150–180g were purchased from Guangdong Medical Laboratory Animal Center (Foshan, China) and maintained in a specific pathogen-free (SPF) animal facility under a 12-h light/12-h dark cycle, with free access to food and water. All rats were subjected to 1week of acclimatization prior to the start of experiment. In Experimental Part I, the rats were randomly divided into the following groups (*n*=7 rats per group): (1) sham group and (2) 5/6 nephrectomy-induced CKD group (5/6 Nx-CKD). The 5/6 Nx operation was performed in accordance with our previous publication (Liu et al., 2018). The sham group only exposed the kidney without destroying the kidney tissue. Twelve weeks after 5/6 Nx operation, the rats were sacrificed and serum and kidney samples were collected for further analysis. In Experimental Part II, the rats were randomly divided into the following groups (*n*=7 rats per group): (1) control group and (2) adenine-induced CKD group (adenine-CKD). Adenine-induced CKD was established by feeding 0.75% *w*/*w* adenine-containing feed for 4weeks (Liu et al., 2019). The control group was fed with normal feed. At the end of experiments, the rats were sacrificed and serum and kidney samples were collected for further analysis.

### Measurement of Serum Creatinine and Blood Urea Nitrogen

The levels of serum creatinine (Scr) were measured by a Creatinine Serum Detection Kit (#SKT-217, StressMarq Biosciences, BC, Canada). Briefly, 25μl of samples, water as the blank, or standards was pipetted into wells in a clear plate. And then, 25μl of Assay Diluent was added to all wells followed by adding 100μl of the StressXpress Creatinine Reagent to each well. Read the optical density generated from each well in a plate reader capable of reading at 490nm in 1min and 30min, respectively. The levels of blood urea nitrogen (BUN) were measured by a Blood Urea Nitrogen Detection Kit (#SKT-213, StressMarq Biosciences, British Columbia, Canada). Briefly, 50μl of samples, water as the blank, or standards was pipetted into wells in a clear plate. Then, 75μl of Color Reagent A and 75μl of Color Reagent B were added to each well in turn. After incubation at room temperature for 30min, read the optical density at 450nm.

### Histological Examination

Rat kidney samples were fixed with 4% paraformaldehyde (pH 7.4) at 4°C overnight, dehydrated in graded alcohols, and embedded in paraffin. Paraffin-embedded rat kidneys were cut into 4-μm sections and stained with periodic acid-Schiff (PAS) and Masson’s trichrome stains to evaluate renal tubular injury and tubulointerstitial fibrosis. Glomerular hypertrophy was evaluated by the measurement of glomerular tuft area in PAS staining using Nikon NIS-Elements BR software version 4.10.00 (Nikon, Japan).

### Measurement of NAD^+^ and NADH

The content of NAD^+^ and NADH in kidney tissue was measured by using a CheKine NAD/NADH Assay Kit (#KTB1020, Abbkine, Wuhan, China), following the manufacturer’s protocol. The frozen kidney cortexes (~20mg/sample) were homogenized with either 100μl NAD^+^ extraction buffer for NAD^+^ determination or 100μl NADH extraction buffer for NADH determination. After heating extracts at 60°C for 5min, 20μl of Assay Buffer and 100μl of the opposite extraction buffer were added to neutralize the extracts. The supernatants were obtained by centrifugation at 14,000rpm for 5min. Then, 40μl of standards or samples was pipetted into wells followed by adding 80μl of Working Reagent per well quickly. After incubation at room temperature for 60min, read optical density at 565nm. The protein concentration of kidney lysate was measured by the Bradford method. The values of NAD^+^ and NADH in the kidney were normalized to the protein concentrations.

### Western Blot

The frozen kidney cortexes were pulverized in liquid nitrogen and homogenized in RIPA buffer (Cell Signaling Technology, Beverly, MA, United States) containing a protease inhibitor cocktail. Equal amounts of total protein from kidney cortex lysates were loaded and electrophoresed through 10% SDS-PAGE gels and were then transferred to nitrocellulose membranes (Millipore, Billerica, MA, United States). After blocking with 5% non-fat milk for 1h at room temperature, the membranes were incubated with primary antibodies against ACMSD (1:2,000), nicotinamide mononucleotide adenylyltransferase 1 (NMNAT1, 1:1,000), neutrophil gelatinase-associated lipocalin (NGAL, 1:1,000; Abcam, Cambridge, MA, United States), nicotinamide phosphoribosyltransferase (NAMPT, 1:2,000), nicotinic acid phosphoribosyltransferase 1 (NAPRT1, 1:1,000), sirtuin 1 (SIRT1, 1:500), SIRT3 (1:500; Proteintech, Wuhan, China), nicotinamide riboside kinase 1 (NRK1, 1:500), NMNAT3 (1:500; Santa Cruz Biotechnology, Santa Cruz, CA, United States), PARP1 (1:500; Cell Signaling Technology, Beverly, MA, United States), CD38 (1:500; Bioss, Beijing, China), quinolinic acid phosphoribosyltransferase (QPRT, 1:500), and β-actin (1:5,000; Sigma-Aldrich, St Louis, MO, United States) at 4°C overnight. The membranes were next incubated with horseradish peroxidase (HRP)-conjugated secondary antibodies (1:2,000; Life Technologies, Carlsbad, CA, United States) and Immobilon Western Chemiluminescent HRP Substrate (Millipore, Billerica, MA, United States). The bands were visualized and analyzed by using ChemiDoc MP Imaging System (Bio-Rad Laboratories, Hercules, CA, United States).

### Statistical Analysis

All data were expressed in mean±standard error of mean (SEM). Statistical differences were examined by Student’s two-tailed unpaired *t*-test. The correlation between NAD^+^ content and renal function indexes was performed by Pearson’s correlation analysis. A value of *p*<0.05 was considered significant differences.

## Results

### The Levels of Renal Injury Indexes in Normal and CKD Rats

**Figure 1:**
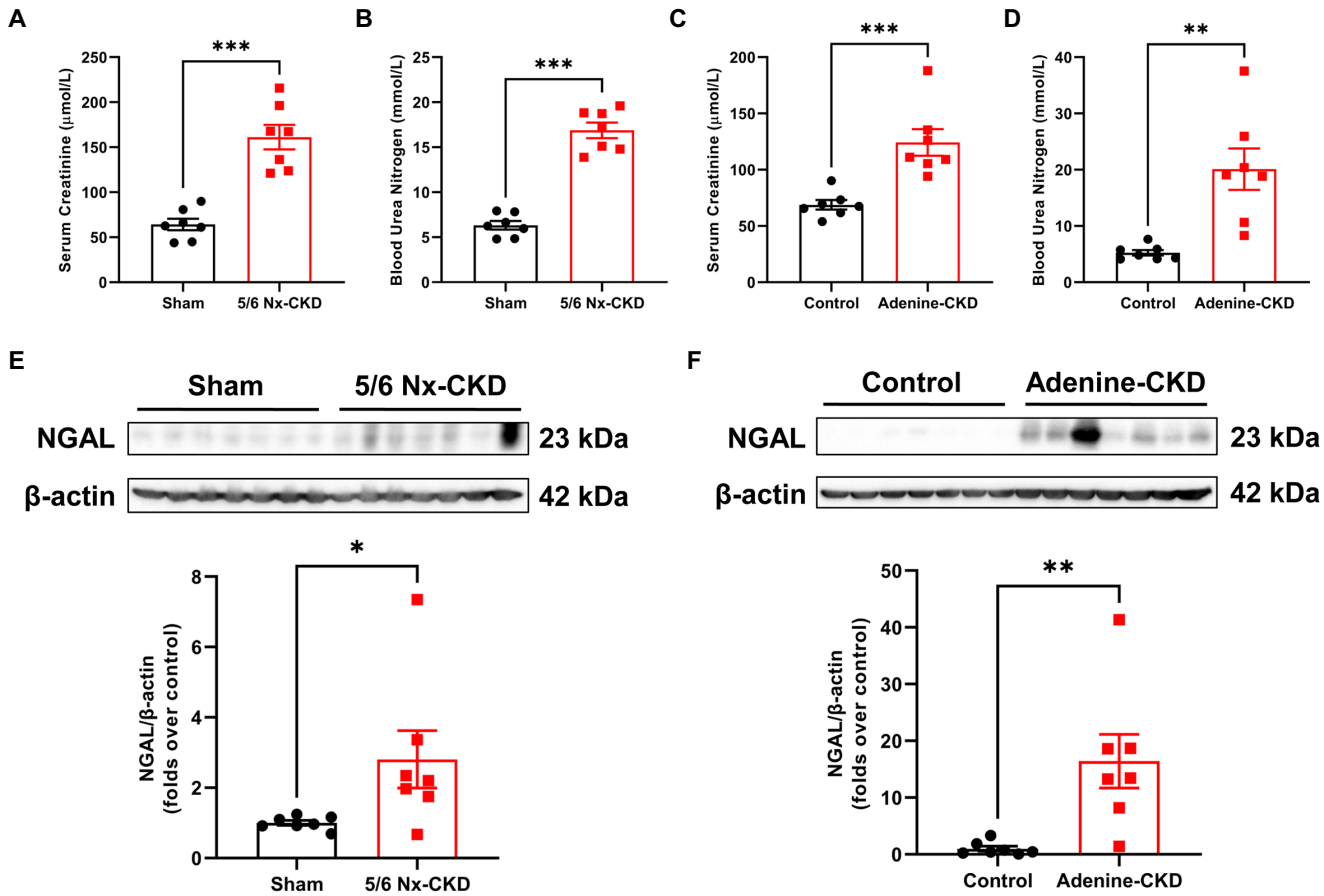
Biochemical analysis of renal function indexes. **(A)** The serum creatinine (Scr) levels in sham and 5/6 Nx-induced chronic kidney disease (CKD) rats. **(B)** The blood urea nitrogen (BUN) levels in sham and 5/6 Nx-induced CKD rats. **(C)** The Scr levels in control and adenine-induced CKD rats. **(D)** The BUN levels in control and adenine-induced CKD rats. **(E)** Western blot images and densitometric analysis of NGAL expression in the kidney of sham and 5/6 Nx-induced CKD rats. **(F)** Western blot images and densitometric analysis of NGAL expression in the kidney of control and adenine-induced CKD rats. Data are presented as the means±SEM, *n*=7 rats per group (^*^ *p*<0.05, ^**^ *p*<0.01, and ^***^ *p*<0.001 between the indicated two groups).

Serum creatinine and BUN are common biomarkers for evaluating renal function. As shown in Figures 1A,B, the mean levels of Scr and BUN in 5/6 Nx-induced CKD rats were 2.5 times and 2.7 times that of sham rats, respectively (*p*<0.001). Similarly, the mean levels of Scr and BUN in adenine-induced CKD rats were 1.8 times and 3.8 times that of control rats, respectively (Figures 1C,D). Since Scr and BUN levels could be affected by different factors including muscle mass and protein intake (Krstic et al., 2016), we further tested the expression of NGAL in the kidney. Western blot analyses found that NGAL was markedly upregulated in CKD rats (Figures 1E,F). These data collectively indicated that CKD rat models were successfully established by both 5/6 Nx and adenine induction.

### Characteristics of Renal Pathology in Normal and CKD Rats

**Figure 2:**
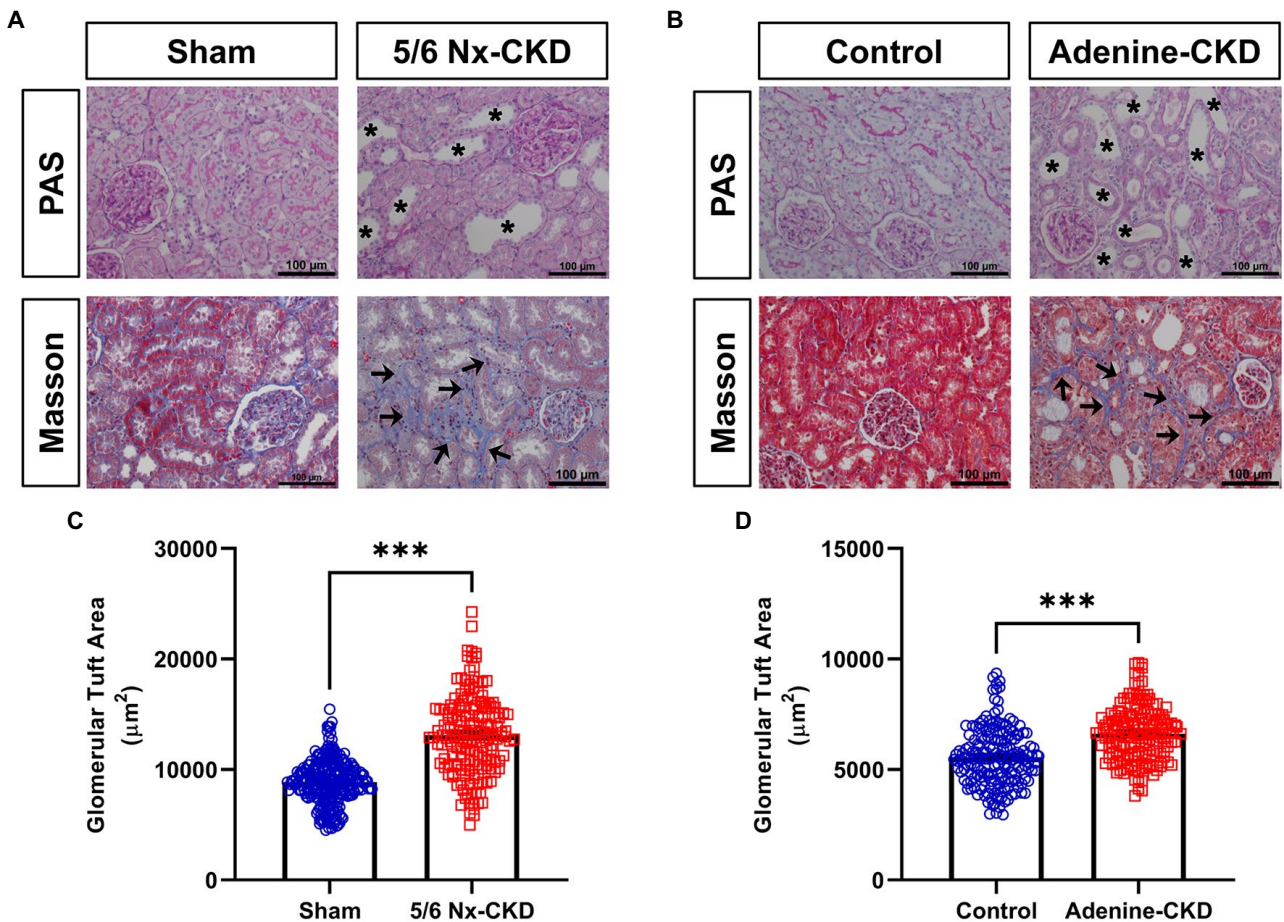
Pathological analysis of kidney tissue structure. **(A)** Representative images of periodic acid-Schiff (PAS) and Masson staining in sham and 5/6 Nx-induced CKD rats. **(B)** Representative images of PAS and Masson staining in control and adenine-induced CKD rats. All images are shown at identical magnification, ×200, scale bar=100μm. Asterisk indicates tubular expansion, and arrow indicates collagen fibril. **(C)** Glomerular tuft area in sham and 5/6 Nx-induced CKD rats. **(D)** Glomerular tuft area in control and adenine-induced CKD rats. Data are presented as the means±SEM, *n*=150–200 glomeruli from four rats per group (^***^ *p*<0.001 between the indicated two groups).

In PAS staining, CKD rats showed renal tubular epithelial cell atrophy, shedding, and tubular lumen dilation. Masson staining displayed obvious accumulation of collagen fibrils (blue staining) in tubulointerstitium of CKD rats. These typical pathological changes were similar in 5/6 Nx and adenine-induced CKD rat models (Figures 2A,B). Glomerular hypertrophy is the feature of CKD. The glomerular tuft area in 5/6 Nx-induced CKD rats was obviously increased compared with the sham group (13,110±272.2μm^2^ vs. 8,864±124.5μm^2^, *p*<0.001; Figure 2C). Due to the relatively short experimental period, adenine-induced CKD only slightly increased the glomerular tuft area (6,619±97.3μm^2^ vs. 5,562±96.6μm^2^, *p*<0.001; Figure 2D).

### The Content of NAD^+^ and NADH in the Kidneys of Normal and CKD Rats

**Figure 3:**
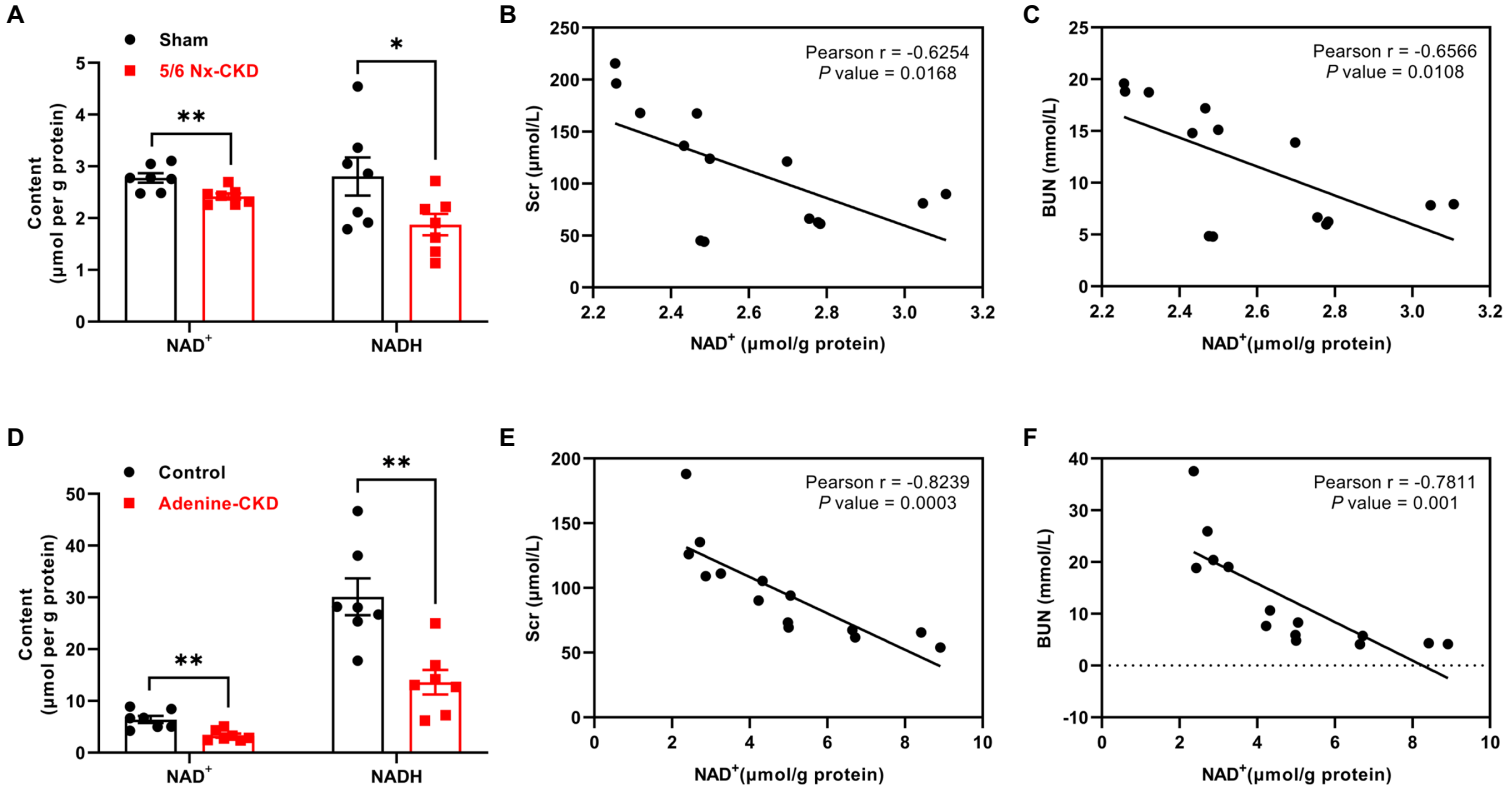
The content of NAD^+^ and NADH in the kidney and the correlation analysis between NAD^+^ levels and renal function indexes. **(A)** The content of NAD^+^ and NADH in the kidney of sham and 5/6 Nx-induced CKD rats. **(B)** The correlation between NAD^+^ and Scr in Experimental Part I (*n*=14). **(C)** The correlation between NAD^+^ and BUN in Experimental Part I (*n*=14). **(D)** The content of NAD^+^ and NADH in the kidney of control and adenine-induced CKD rats. **(E)** The correlation between NAD^+^ and Scr in Experimental Part II (*n*=14). **(F)** The correlation between NAD^+^ and BUN in Experimental Part II (*n*=14). In **(A)** and **(D)**, data are presented as the means±SEM, *n*=7 rats per group (^*^ *p*<0.05 and ^**^ *p*<0.01 between the indicated two groups).

Compared with the sham group, renal NAD^+^ and NADH content decreased by 12.9 and 33.1%, respectively, in 5/6 Nx-induced CKD rats (*p*<0.01 and 0.05, respectively; Figure 3A). In Experimental Part I, NAD^+^ levels were negatively correlated with Scr and BUN levels, and the correlation coefficients were −0.6254 and −0.6566, respectively (*p*<0.05; Figures 3B,C). Similarly, in adenine-induced CKD rats, renal NAD^+^ and NADH content were also significantly decreased (*p*<0.01; Figure 3D), and NAD^+^ levels were also negatively correlated with Scr and BUN levels (*r*=−0.8239 and −0.7811, respectively, *p*<0.01; Figures 3E,F).

### The Expression of Related Enzymes in NAD^+^ Biosynthesis Pathways

**Figure 4:**
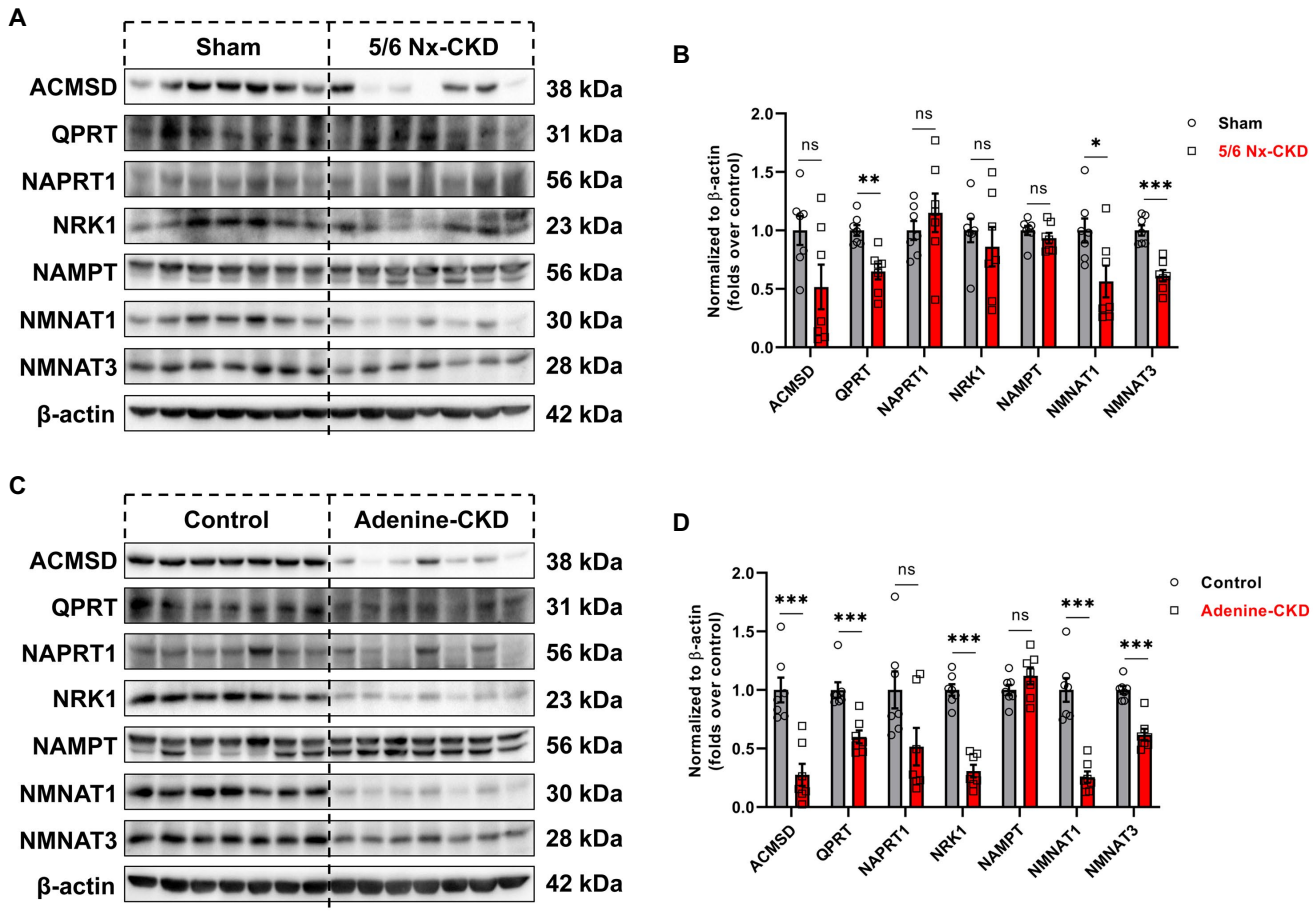
The expression of related enzymes in the NAD^+^ biosynthesis pathways. **(A)** Representative Western blot images of 7 enzymes in the kidney of sham and 5/6 Nx-induced CKD rats. **(B)** Densitometric analysis of 7 enzymes expression in the kidney of sham and 5/6 Nx-induced CKD rats, normalized to β-actin content. **(C)** Representative Western blot images of 7 enzymes in the kidney of control and adenine-induced CKD rats. **(D)** Densitometric analysis of 7 enzymes expression in the kidney of control and adenine-induced CKD rats, normalized to β-actin content. Data are presented as the means±SEM, *n*=7 rats per group (^*^ *p*<0.05, ^**^ *p*<0.01, and ^***^ *p*<0.001 between the indicated two groups).

In Experimental Part I, the protein abundance of QPRT, NMNAT1, and NMNAT3 was significantly decreased in the 5/6 Nx-CKD group. There were no statistical differences in the expression of other enzyme proteins between the sham group and the 5/6 Nx-CKD group (Figures 4A,B). In Experimental Part II, except for NAPRT1 and NAMPT, the expression of ACMSD, QPRT, NRK1, NMNAT1, and NMNAT3 was all significantly downregulated in adenine-induced CKD rats (Figures 4C,D).

### The Expression of NAD^+^-Consuming Enzymes in the Kidneys of Normal and CKD Rats

**Figure 5:**
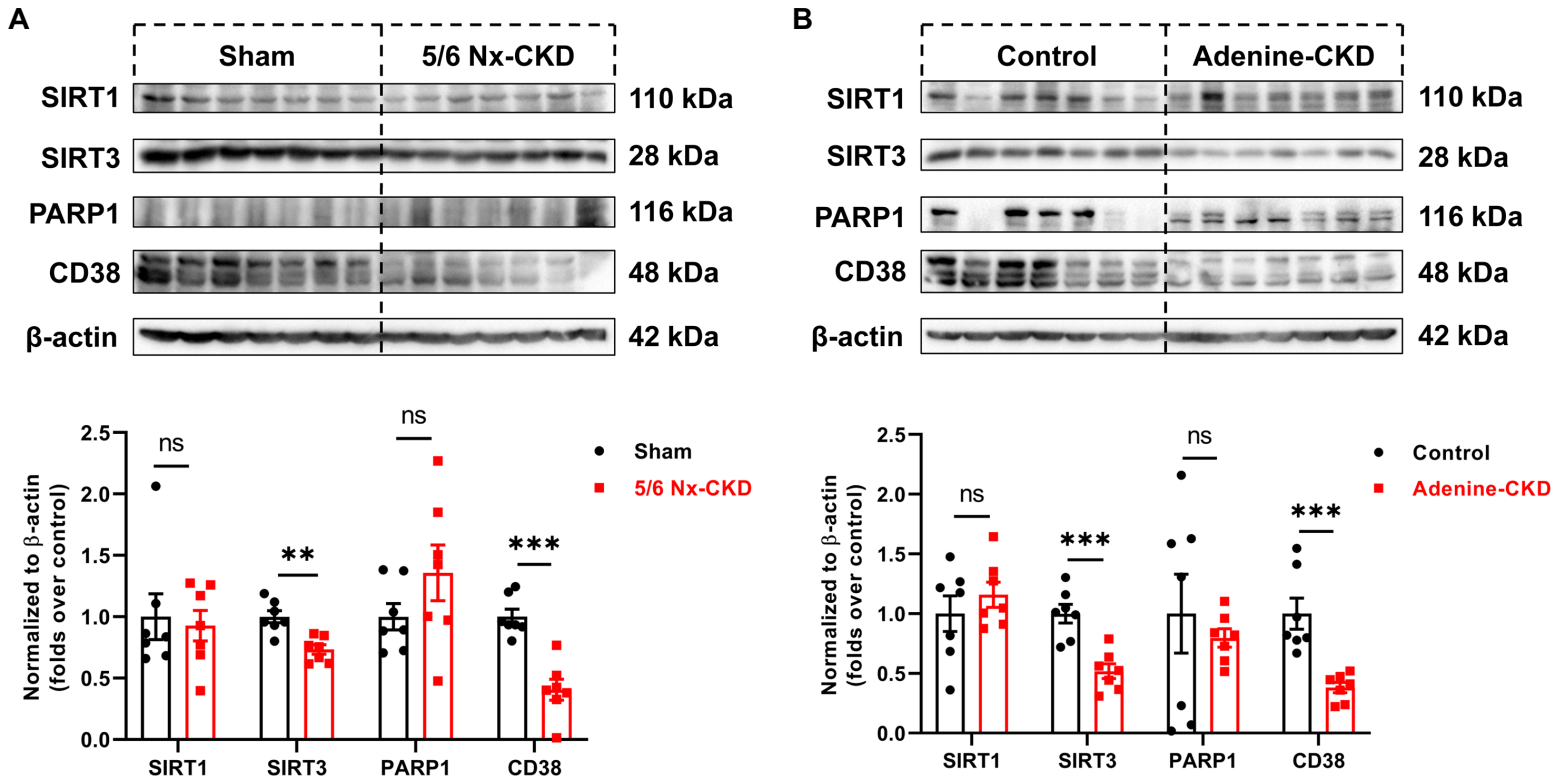
The protein expression of NAD^+^-consuming enzymes. **(A)** Representative Western blot images and densitometric analysis of SIRT1, SIRT3, PARP1, and CD38 in the kidney of sham and 5/6 Nx-induced CKD rats. **(B)** Representative Western blot images and densitometric analysis of SIRT1, SIRT3, PARP1, and CD38 in the kidney of control and adenine-induced CKD rats. Data are presented as the means±SEM, *n*=7 rats per group (^**^ *p*<0.01 and ^***^ *p*<0.001 between the indicated two groups).

In the salvage pathway, NAD^+^ synthesis can occur through recycling of NAM, which is generated by NAD^+^-consuming enzymes such as sirtuins, PARP, and CD38. The expression of major NAD^+^ consumers of SIRT1, SIRT3, PARP1, and CD38 was measured by Western blot. As shown in Figures 5A,B, SIRT3 and CD38 were markedly downregulated in CKD rats by both 5/6 Nx and adenine induction (*p*<0.01). Compared with normal rats, the contents of SIRT1 and PARP1 in the kidneys of both 5/6 Nx and adenine-induced CKD rats did not change significantly.

## Discussion

**Figure 6:**
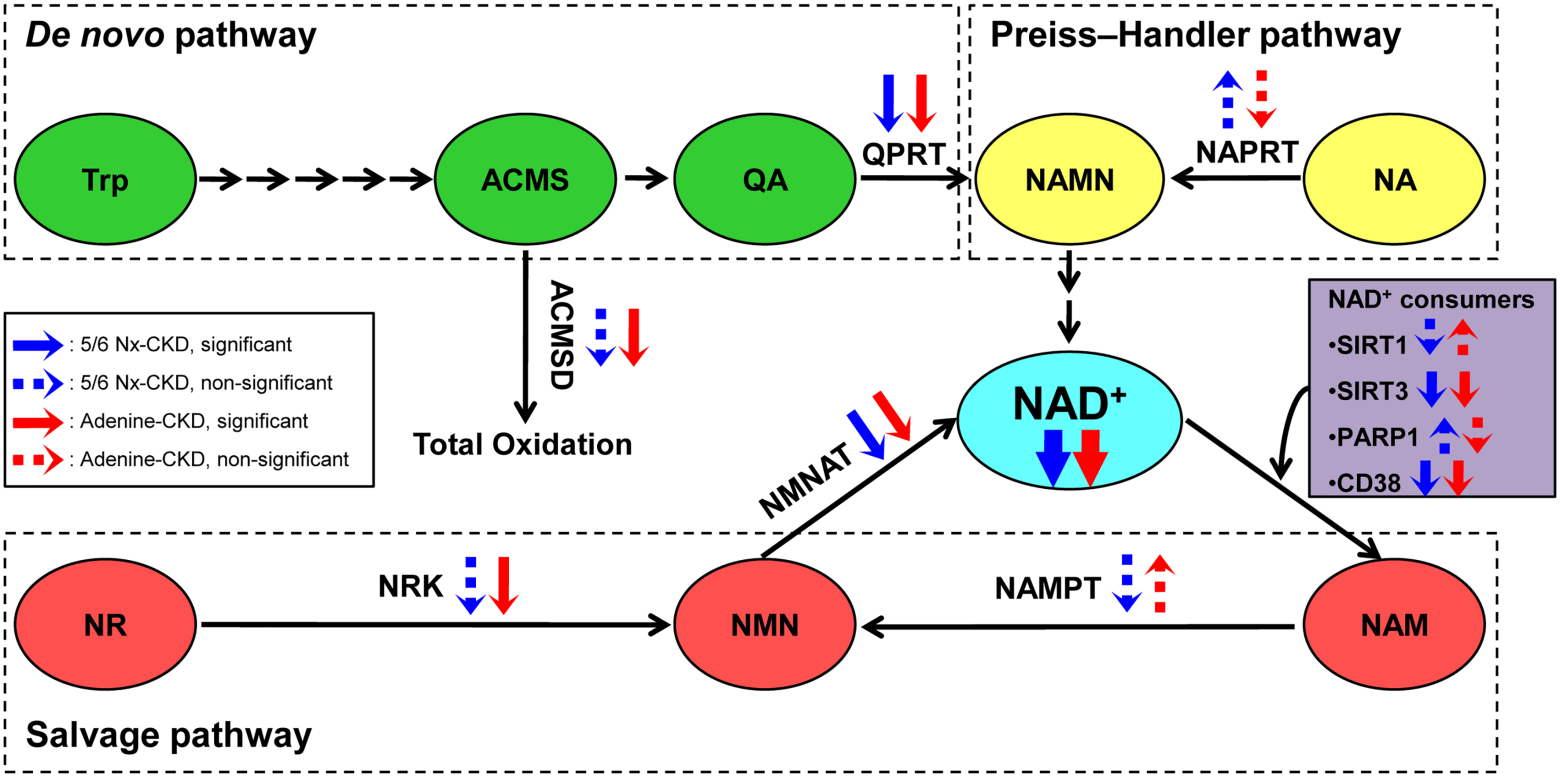
The scheme summary of alterations in the three NAD^+^ biosynthesis pathways in the setting of CKD. ACMS, α-amino-β-carboxymuconate-ε-semialdehyde; ACMSD, α-amino-β-carboxymuconate-ε-semialdehyde decarboxylase; NA, nicotinic acid; NAD, nicotinamide adenine dinucleotide; NAM, nicotinamide; NAMN, nicotinic acid mononucleotide; NAMPT, nicotinamide phosphoribosyltransferase; NAPRT, nicotinic acid phosphoribosyltransferase; NMN, nicotinamide mononucleotide; NMNAT, nicotinamide mononucleotide adenylyltransferase; NR, nicotinamide riboside; NRK, nicotinamide riboside kinase; PARP1, poly (ADP-ribose) polymerase 1; QA, quinolinic acid; QPRT, quinolinic acid phosphoribosyltransferase; SIRT1, sirtuin 1; SIRT3, sirtuin 3; and Trp, tryptophan.

In the present study, we successfully established two different types of CKD rat models. We found that renal NAD^+^ and NADH content were both declined in two CKD rat models, and NAD^+^ levels were negatively correlated with Scr and BUN levels in CKD rats. Three key enzymes involved in NAD^+^ biosynthesis were significantly downregulated in the kidney of both of the two CKD models. They were QPRT in the *de novo* pathway, NMNAT1 and NMNAT3 in the salvage pathway. In addition, SIRT3 and CD38, major NAD^+^-consuming enzymes, were markedly downregulated in CKD rats (Figure 6).

NAD^+^ is a ubiquitous coenzyme involved in electron transport in mitochondria and an essential cofactor for sirtuins and poly-adenosine triphosphate ribose polymerases (Covarrubias et al., 2021). Decreased NAD^+^ levels were found to be associated with aging, cancer, and neurodegenerative and metabolic disorders (Amjad et al., 2021). Recent studies have also implicated the essential role of NAD^+^ in maintaining kidney health. Supplementation with nicotinamide mononucleotide (NMN), an NAD^+^ precursor, rescued age-associated susceptibility to AKI in 20-month-old mice (Guan et al., 2017). Oh et al. (2014) reported that pharmacological activation of quinone oxidoreductase 1 (NQO1) by β-lapachone increased NAD^+^ levels and attenuated cisplatin-mediated AKI in mice. Nicotinamide (NAM), a member of water-soluble vitamin B family, is a key precursor for the biosynthesis of NAD^+^ *via* salvage pathway. In unilateral urethral obstruction (UUO) mice model, NAM suppressed tubular atrophy, inflammation, and fibrosis (Zheng et al., 2019). In adenine-induced CKD mice model, NAM supplementation also reduced kidney inflammation and fibrosis and prevented the progression of kidney disease (Kumakura et al., 2021). Diabetic nephropathy (DN) is the leading cause of CKD worldwide (Alicic et al., 2017). Short-term NMN treatment in early-stage DN had remote renal protective effects through the upregulation of Sirt1 and increase NAD^+^ levels (Yasuda et al., 2021). These studies collectively indicated that decreased NAD^+^ level was common in AKI and CKD, and boosting NAD^+^ biosynthesis could delay the progression of kidney disease. The 5/6 Nx is a surgical ablation model widely used to study the progression of CKD. Due to the loss of renal mass, the residual nephrons undergo changes in metabolism and hemodynamics, which ultimately lead to compensatory hypertrophy of the remnant kidney (Aparicio-Trejo et al., 2017). The addition of 0.75% adenine to the diet of rats for 4weeks gained general acceptance as this intervention mimicked most of the structural and functional changes seen in human CKD (Diwan et al., 2018). After intake, adenine is oxidized to form 2,8-dioxyadenine, which is deposited as crystalline occlusion in renal tubules (Philips et al., 1952). Our study confirmed the deficiency of NAD^+^ in these two different types of CKD rat models and provided a basis for future drug intervention research.

NAD^+^ biosynthesis can be achieved from tryptophan through *de novo* pathway or from precursors such as NAM, NMN, NR, or NA (Cantó and Auwerx, 2012). Recently, two studies demonstrated that alteration of *de novo* pathway played an important role in AKI. QPRT, a bottleneck enzyme in *de novo* biosynthesis, defended renal NAD^+^ and mediated resistance to AKI (Poyan Mehr et al., 2018). ACMSD inhibition improved mitochondrial function, increased NAD^+^, and prevented AKI in mice (Katsyuba et al., 2018). However, the alteration of NAD^+^ biosynthesis pathways in CKD is less known. Our results showed that QPRT and NMNAT 1/3 were significantly downregulated in both of the two CKD models, which indicated that the *de novo* and the salvage pathway were both impaired in CKD. NMNAT is responsible for converting NMN to NAD^+^. NMNAT 1 is localized in the nucleus and possesses the most robust enzymatic activity among the three NMNAT isozymes (Raffaelli et al., 2002; Berger et al., 2005). NMNAT 3 is localized in the mitochondria and responsible for maintaining mitochondrial NAD^+^ homeostasis (Zhang et al., 2003; VanLinden et al., 2015). Most of the previous studies were focused on supplementation NAD^+^ precursors to boost biosynthesis *via* salvage pathway. This strategy may not be effective in the CKD model. Faivre et al. (2021) reported that NR supplementation was beneficial in ischemic AKI but not in CKD models. This study also found that NAD^+^ *de novo* synthesis pathway was impaired according to CKD stage, with better preservation of the salvage pathway in human biopsies from CKD patients (Faivre et al., 2021). Similar to this study, our data also suggested that impaired NAD^+^ *de novo* synthesis was important in CKD. Therefore, it may be meaningful to develop new drugs to promote NAD^+^ *de novo* synthesis for CKD treatment.

The limitation of the present study was that we did not verify our results by regulating the expression of key enzymes or supplementation of NAD^+^ precursors. The current conclusions about the role of NAD^+^ precursor supplementation in CKD are inconsistent. Kumakura et al. (2021) demonstrated that NAM attenuated the progression of renal failure in a mouse model of adenine-induced CKD, while NR was found to not prevent CKD progression in UUO or chronic proteinuria (POD-ATTAC) mice models (Faivre et al., 2021). Both NAM and NR are first converted to NMN, and then, NAD^+^ is synthesized under the catalysis of NMNATs. Our results revealed that NMNAT 1/3 was downregulated in CKD models. Therefore, supplementing NMN or upregulating NMNAT 1/3 expression may be beneficial to delay the progression of CKD. In addition, QPRT, key enzyme in NAD^+^ *de novo* synthesis, was found to be significantly downregulated in CKD models. To our knowledge, there is currently no specific agonist for QPRT, which requires further pharmacological studies.

In conclusion, renal NAD^+^ levels were decreased and negatively correlated with renal function indexes in two different types of CKD rat model. Deficient NAD^+^ biosynthesis may attribute to downregulation of QPRT in the *de novo* pathway and NMNAT 1/3 in the salvage pathway.

## Data Availability Statement

The original contributions presented in the study are included in the article/supplementary material, further inquiries can be directed to the corresponding authors.

## Ethics Statement

The animal study was reviewed and approved by the Experimental Animal Ethics Committee of Guangzhou University of Chinese Medicine.

## Author Contributions

XL and SLi conceived and designed the experiments. DL, SH, SLiu, and FW carried out animal experiment and conducted the pathological analysis. BZ and JL contributed to data collection and manuscript review. XL and JC analyzed the data, prepared the figures, and wrote the manuscript. All authors contributed to the article and approved the submitted version.

## Funding

This study was supported by Natural Science Foundation of China (81973602 and 81804052), Shenzhen Science and Technology Plan Project (JCYJ20190812161001790, JSGG20191129102216637, JCYJ20180302173708520, and JCYJ20180507183842516), and Natural Science Foundation of Guangdong Province (2020A1515011151 and 2018A030313305).

## Conflict of Interest

The authors declare that the research was conducted in the absence of any commercial or financial relationships that could be construed as a potential conflict of interest.

## Publisher’s Note

All claims expressed in this article are solely those of the authors and do not necessarily represent those of their affiliated organizations, or those of the publisher, the editors and the reviewers. Any product that may be evaluated in this article, or claim that may be made by its manufacturer, is not guaranteed or endorsed by the publisher.
